# Modeling of Solute-Solvent Interactions Using an External Electric Field—From Tautomeric Equilibrium in Nonpolar Solvents to the Dissociation of Alkali Metal Halides

**DOI:** 10.3390/molecules26051283

**Published:** 2021-02-26

**Authors:** Ilya G. Shenderovich, Gleb S. Denisov

**Affiliations:** 1 Institute of Organic Chemistry, University of Regensburg, Universitaetstrasse 31, 93053 Regensburg, Germany; 2 Department of Physics, St. Petersburg State University, 198504 St. Petersburg, Russia; gldenisov@yandex.ru

**Keywords:** solvent effect, dissociation, tautomerism, condensed matter, polarizable continuum model, reaction field, external electric field

## Abstract

An implicit account of the solvent effect can be carried out using traditional static quantum chemistry calculations by applying an external electric field to the studied molecular system. This approach allows one to distinguish between the effects of the macroscopic reaction field of the solvent and specific solute–solvent interactions. In this study, we report on the dependence of the simulation results on the use of the polarizable continuum approximation and on the importance of the solvent effect in nonpolar solvents. The latter was demonstrated using experimental data on tautomeric equilibria between the pyridone and hydroxypyridine forms of 2,6-di-tert-butyl-4-hydroxy-pyridine in cyclohexane and chloroform.

## 1. Introduction

Noncovalent interactions allow our world to exist. Their specific interaction energies [1,2,3,4], their effect on the spectral [5,6,7], chemical [8,9], and structural [10,11,12] properties of individual molecules, those in adducts and solutions [13,14,15], have been and remain vital to the progress of chemistry. The interpretation of spectral shifts caused by changes in the properties of a particular interaction can be carried out in sufficient detail [16]. In contrast, the result of a complex interplay of competing noncovalent interactions in condensed matter is often challenging to predict, even in crystalline solids in which the positions of atoms are known and do not change with time [17,18,19,20,21,22,23,24]. In the case of solutions, the situation becomes even more complicated. Small changes of one interaction lead to measurable changes in others. Correct interpretation of these changes requires the selection of a convenient model system and a preliminary study of the dependence of the discussed spectral characteristic on the interaction properties [25,26]. In other cases, only a general interpretation of the observed spectral changes caused by changing noncovalent interactions is possible [27,28].

The situation in which the properties of the molecular system under consideration depend on its interaction with the solvent is most common. The best method for such cases is the molecular dynamics approach [29,30,31]. Even here, simplified solvation models are often used [32,33,34]. However, such calculations are still laborious and difficult to apply both for large molecular systems and for qualitative model calculations. Less demanding approaches are the polarizable continuum model (PCM) [35,36,37] and solvation model based on density (SMD) [38] approximations. These approaches significantly improve the description of molecular systems in solutions. However, it has been clearly demonstrated that, at least for highly polar solvents, both of these approaches underestimate the effect of solvation [39]. The agreement with experimental results can be achieved either by changing the geometry of the molecular system in the “correct direction” [40], by introducing specially selected additional intermolecular interactions [41], or by applying an external electric field—the Adduct under Field (AuF) approach [42,43]. The latter approach is particularly simple and robust. The macroscopic electric field generated by the dipole moments of the solvent molecules and weak yet multiple interactions with the solvent molecules cause changes in the electron density of the solvated molecules. These changes can be simulated using the external electric field of a selected strength. This approach has been successfully used in the past to study dependencies between the properties of the electron distribution, molecular properties, and intermolecular interactions [44,45,46,47,48,49,50,51,52,53,54]. For some molecular systems, the strength of this field has been experimentally estimated [55,56]. The combination of the PCM approach and the external electric field appears to provide more reliable and stable results than the use of the external electric field under the gas-phase approximation [43,56]. This aspect needs to be purposefully studied in order to help with an accurate prediction of this approach. Another critical question is how accurately the PCM and SMD approaches reproduce the effects of solvation in nonpolar solvents.

**Figure 1:**
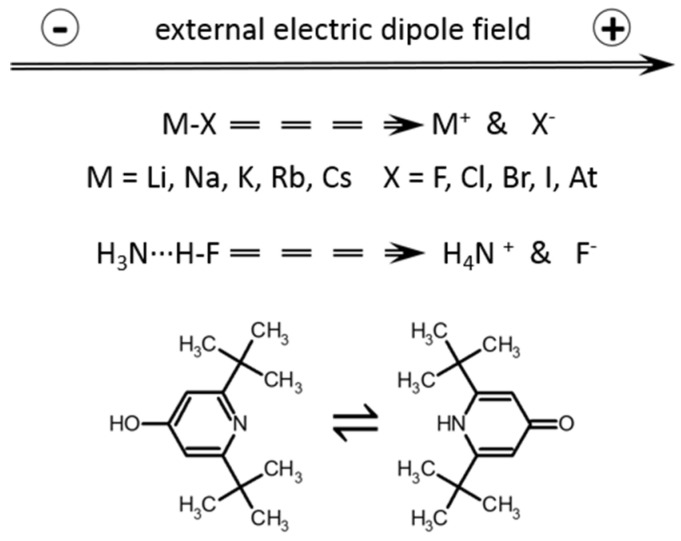
Molecular systems studied in this work and the direction of the external electric field acting on them.

In order to answer the first question, the dissociation of alkali metal halides, MX, and proton transfer in a hydrogen bonded H_3_N···HF complex under the action of an external electric field has been studied in the gas-phase and PCM approximations (Figure 1). This study aims to explore which of these two approaches can be recommended for future use. The second problem has been studied using the experimental data on the pyridone–hydroxypyridine equilibria in nonpolar solvents [57] (Figure 1). The purpose of this part is to find out if the experimental results are reproducible within the PCM and SMD approximations, and if it is possible to improve the agreement with the experiment using an external electric field. Note that the effect of an external electric field on chemical equilibrium was demonstrated experimentally [58,59,60].

## 2. Results and Discussion

### 2.1. Alkali Metal Halides

**Figure 2:**
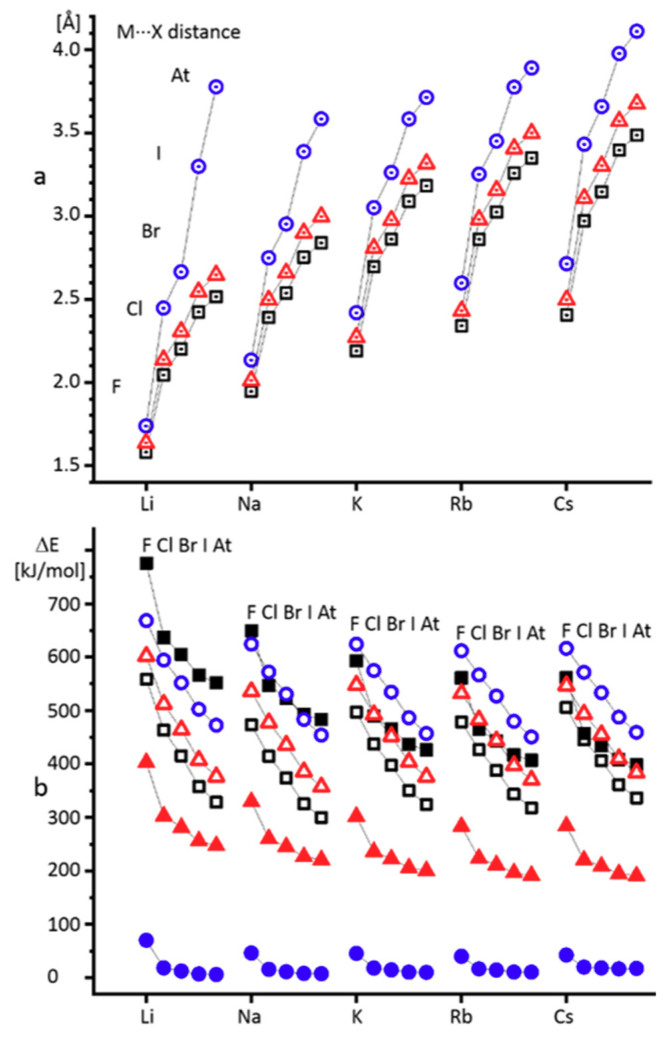
(**a**) Alkali metal···halogen distances under the gas-phase approximation (ε = 1, black squares) and the polarizable continuum model (PCM) approximation at ε = 2 (red triangles) and ε = 100 (blue circles). (**b**) Alkali metal halide dissociation energies under the gas-phase approximation into atoms (empty black squares) and ions (filled black squares), the PCM approximation at ε = 2 into atoms (empty red triangles) and ions (filled red triangles) and at ε = 100 into atoms (empty blue circles) and ions (filled blue circles).

Figure 2a shows the dielectric dependence of the M···X distance in alkali metal halides. Numerical data are collected in Tables S1–S3 in the Supporting Information. All complexes exhibit the expected elongation of the distance in a polar environment. The complex does not dissociate, because there is no interaction that could lead to this. Figure 2b shows the energy required for the dissociation of these complexes into neutral atoms or cation–anion pairs. Numerical data are collected in Tables S1–S5 in the Supporting Information.

In the gas phase, the dissociation into the neutral atoms is energetically more favorable than into the ion pairs. However, the situation is reversed even at a very low dielectric constant of 2. At a dielectric constant of 100, the dissociation into the ion pairs requires significant energy only for alkali metal fluorides (Figure 2b). It is noteworthy that all complexes demonstrate a qualitatively similar dependence of the geometric and energy parameters on the dielectric constant. Further, only LiF, LiAt, KBr, CsF, and CsAt will be studied.

**Figure 3:**
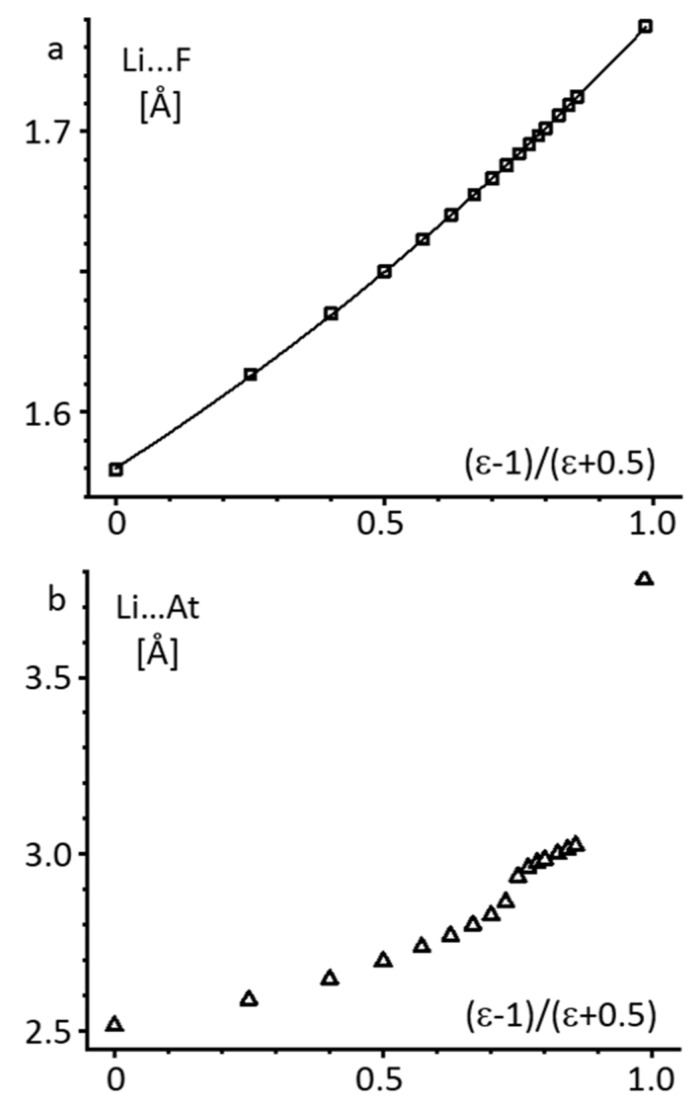
The alkali metal···halogen distance in LiF (**a**) and LiAt (**b**) as a function of (ε − 1)/(ε + 0.5) under the PCM approximation at 1 ≤ ε = ≤ 100.

Figure 3a shows the functional dependence of the Li···F distance on (ε − 1)/(ε + 0.5). The dependence is not perfectly linear, but is monotonic and has no points of inflection. Similar dependences are observed for KBr and CsAt (Figure S1a,c, Supporting Information). In contrast, for LiAt and CsF the dependencies have no points of inflection for ε ≤ 5 only (Figure 3b and Figure S1b). Numerical data are collected in Table S6 in the Supporting Information. While the numerical values of the Li···At distance depend on the basis set used, the general trend does not (Figure S2 and Table S7). Therefore, the results obtained for LiAt and CsF at ε > 5 should be treated with caution.

**Figure 4:**
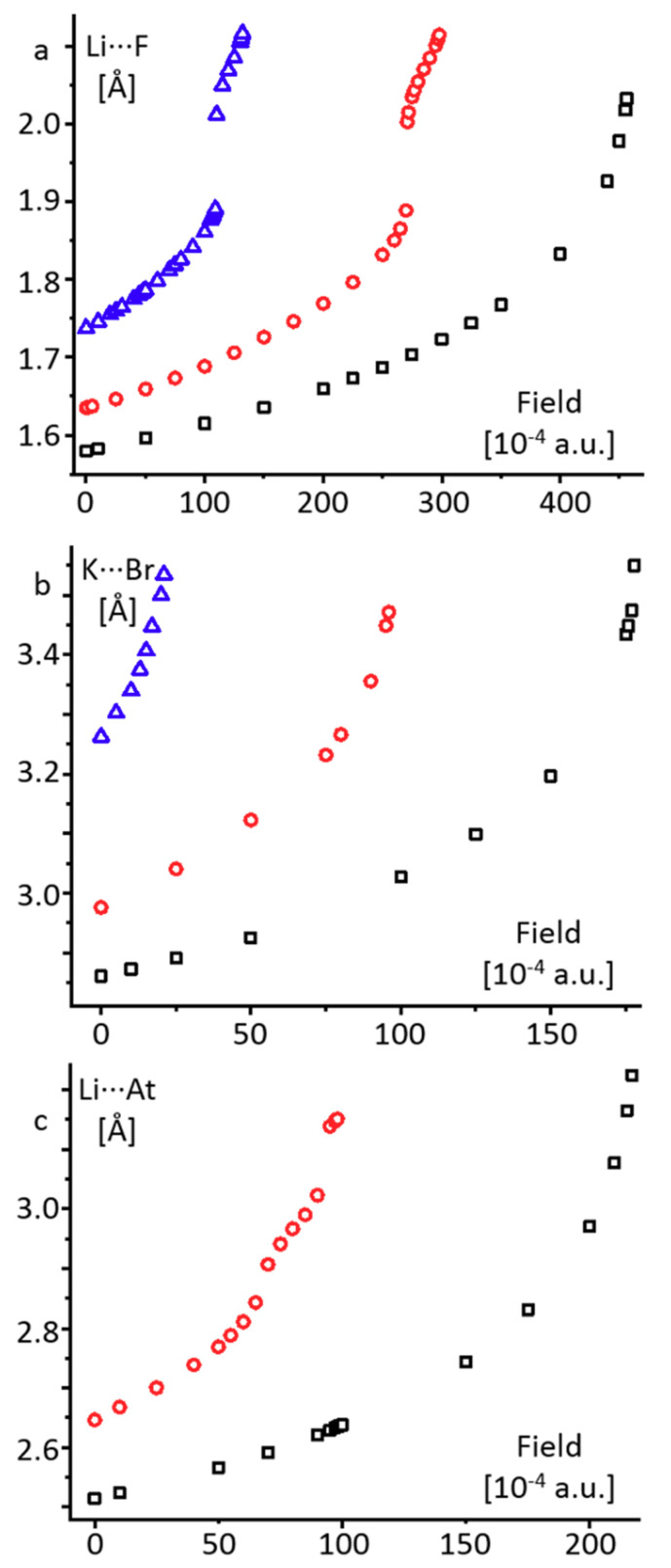
The alkali metal···halogen distance in LiF (**a**), KBr (**b**), and LiAt (**c**) as a function of the external electric field under the gas-phase (ε = 1) and PCM (ε = 2 and 100) approximations.

Figure 4 shows the functional dependencies of the alkali metal···halogen distance on the external electric field at different dielectric constants for selected complexes. Numerical data for all studied complexes are collected in Tables S8–S12 in the Supporting Information. In the gas-phase approximation (ε = 1) these dependencies have no points of inflection up to the dissociation limit of the complexes. An increase in the field strength by 0.0001 a.u. above this limit causes the dissociation of the given alkali metal halide. Under the PCM approximation (ε ≥ 2), the profiles of the dependencies for LiF and LiAt have no points of inflection for short distances only, that is, below 1.90 Å and 2.85 Å (Figure 4a,c, respectively). For KBr the dependence has no points of inflection over the entire distance interval. In this range of distances, the correlations between the external electric fields required to obtain the same alkali metal···halogen distance in the gas-phase (ε = 1) and PCM (ε ≥ 2) approximations are close to linear (Figures S3–S5 in the Supporting Information). Therefore, if one is only interested in comparing the effect of an external field on the alkali metal···halogen distance in different complexes well below the dissociation limit, there is no difference in using the gas-phase or PCM approximation.

The change of the profile for LiAt at longer distances resembles the dependence of the distance on (ε − 1)/(ε + 0.5) (Figure 3b). In both cases changes happen close to 2.9 Å and above 3.0 Å (Figure 3b and Figure 4c). Presumably, for LiF the same effect for the dependence of the distance on (ε − 1)/(ε + 0.5) cannot be observed because the Li···F distance is shorter than 1.9 Å even at ε = 100 (Figure 3a). Note that at the points of inflection the functions exhibit jumps, that is, an increase in the field strength by 0.0001 a.u. leads to a significant increase in the distance (Figure 4a,c).

**Figure 5:**
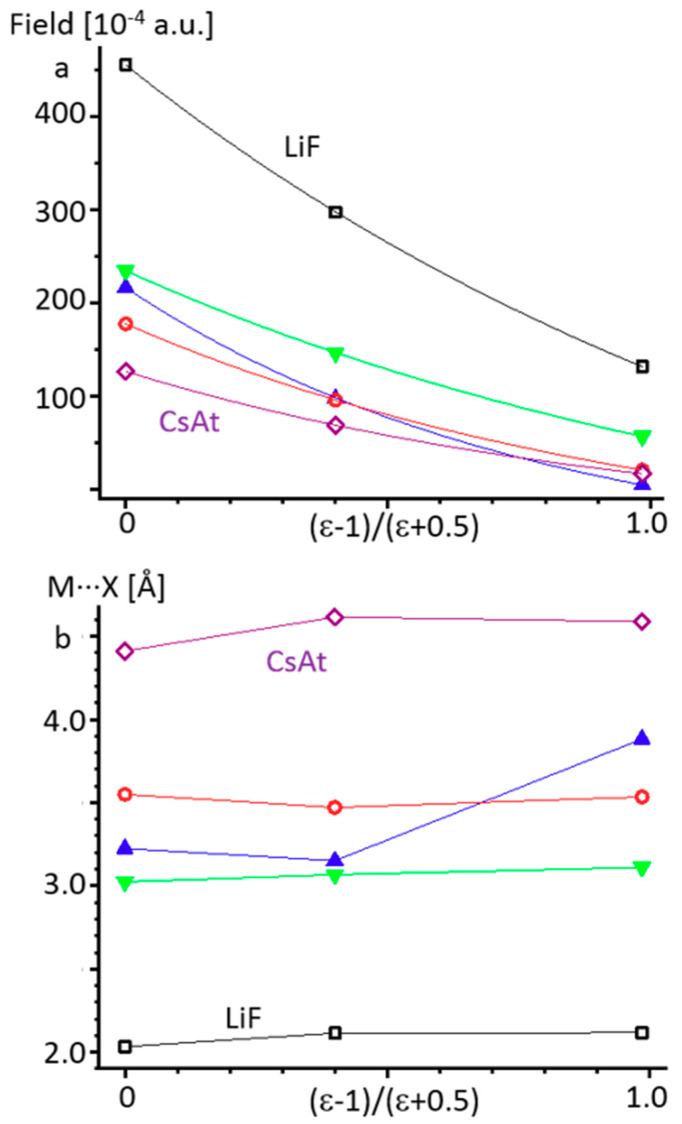
The limiting values of the external electric field (**a**) and the alkali metal···halogen distances (**b**) before the dissociation of LiF (empty black squares), KBr (empty red circles), CsAt (empty lilac diamonds), LiAt (blue upward-pointing triangles), and CsF (green downward-pointing triangles) as a function of (ε − 1)/(ε + 0.5) under the gas-phase (ε = 1) and PCM (ε = 2 and 100) approximations. Lines are provided as a guide for the reader’s eyes.

Figure 5a shows the limiting values of the external electric field above which alkali metal halides dissociate in the gas-phase and PCM approximations. Numerical data are collected in Table S13 in the Supporting Information. Obviously, the result critically depends on the approximation used. In contrast, the limiting values of the M···X distances before the dissociation are similar for the different conditions, except in the case of LiAt (Figure 5b and Table S13). Consequently, if the simulation of the integral strength of intermolecular interactions leading to the dissociation of alkali metal halides is carried out by applying an external electric field, the result critically depends on the approximation used. If one studies the change of the M···X distance caused by these interactions, the result will be similar at any approximation.

### 2.2. H_3_N···HF Complex

**Figure 6:**
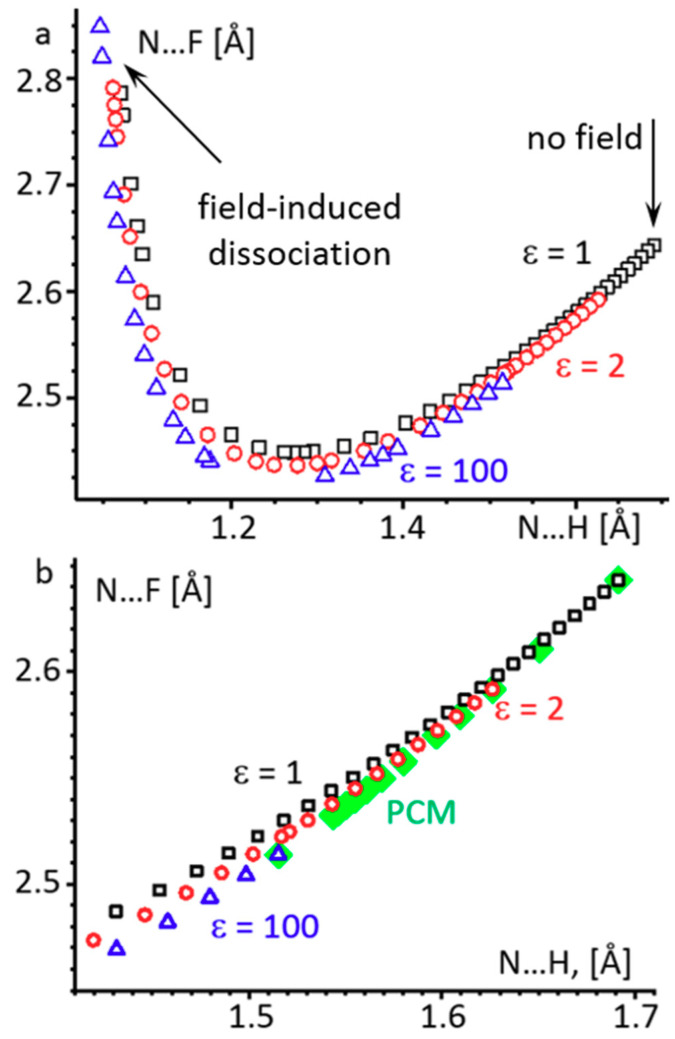
(**a**) A correlation between the H_3_N···H and N···F distances of H_3_N···HF under the effect of an external electric field at the gas-phase (ε = 1, black squares) and PCM (ε = 2, red circles and ε = 100, blue triangles) approximations. (**b**) This correlation for the region of small hydrogen bond perturbation, including the PCM approximation region (green squares).

Figure 6a shows a correlation between the H_3_N···H and N···F distances of H_3_N···HF under the effect of an external electric field at the gas-phase (ε = 1) and PCM (ε = 2 and 100) approximations.

The fact that the A–H and H···B distances of a hydrogen-bonded complex A–H···B are interdependent is well known [61,62,63,64]. The hydrogen bonded H_3_N···HF complex is no exception [40,45,65,66]. In our calculations an increase of the external electric field induces an additional dipole moment in the complex. This induced dipole moment is created by a displacement of the mobile H^+^ toward the nitrogen atom. This proton transfer is accompanied at first by a compression of the N···F distance and then by an elongation of it. Numerical data are collected in Table S14 in the Supporting Information. The first part is associated with the strengthening of the hydrogen bond, while the second one, with its weakening, is followed by dissociation into H_4_N^+^ and F^−^. During the first part of the proton transfer, the difference in the correlations obtained under different conditions is small. This difference is best seen in Figure 6b, where the external field effect is compared with the polarity effect (Table S15). What is particularly important is that the difference between the gas-phase approximation and nonpolar media is very similar to the difference between nonpolar and highly polar media. Therefore, the accuracy of the values of ε used in the PCM approximation is less important than the use of this approximation itself.

The situation is different after the proton is transferred to the base and the hydrogen bond begins to weaken, N···H < 1.2 Å (Figure 6a). Here the correlation between the N···H and N···F distances critically depends on the approximation used. For example, for N···H = 1.083 Å the N···F distance varies from 2.70 Å at ε = 1 to 2.65 Å at ε = 2 to 2.59 Å at ε = 100 (Table S14). The limiting values of these distances before dissociation also change depending on the approximation used. These values for the N···H and N···F distances are 1.071 Å and 2.786 Å, 1.062 Å and 2.791 Å, and 1.047 Å and 2.848 Å at ε = 1, 2, and 100, respectively (Table S14).

Consequently, when modeling the integral effect of intermolecular interactions on the geometry of a hydrogen bonded complex in condensed media is carried out by applying an external electric field, the result depends on whether the PCM approximation is used or not. If the perturbation of the complex is small, the accuracy of the values of ε used in the PCM approximation is not very important. If the electronic structure of the complex changes qualitatively (proton transfer), the difference associated with the value of ε increases noticeably.

### 2.3. Pyridone–Hydroxypyridine Equilibria

The solvent effect on the tautomeric equilibria between pyridone and hydroxypyridine has been extensively studied [57,67,68,69,70]. Hydroxypyridine (OH-form) exists predominantly in the gas phase and in nonpolar solvents, while pyridone (NH-form) is stable in polar solvents. The most pronounced change in the tautomeric equilibrium constant was observed for 2,6-di-tert-butyl-4-hydroxy-pyridine. The tautomeric equilibrium constant for this [NH]/[OH] equilibrium, log([NH]/[OH]), is −0.92 in cyclohexane and 0.96 in chloroform [57]. Assuming that the difference between the zero-point energies of the NH- and OH-forms is negligible, one can associate this constant with the difference of the electronic energies of these forms, Table 1. The gas-phase approximation agrees with the experimental trend—the OH-form is the lowest energy one. The PCM and SMD approximations are also consistent with the experimental trend, but they clearly overestimate the stabilization of the NH-form in solution (Table 1). Presumably, this deviation can be explained by the fact that the NH moiety of 2,6-di-tert-butyl-4-hydroxy-pyridine is sterically protected from specific solute–solvent interactions [71]. In contrast, the OH moiety is not. Consequently, these interactions increase the stabilization of the OH-form in solution.

First we simulate the effect of the intermolecular interactions by applying an external electric field under the gas-phase approximation. The direction of this field is shown in Figure 1. The experimental values of the tautomeric equilibrium constant in cyclohexane and chloroform can be reproduced at a field strength of 0.0007 a.u. and 0.0023 a.u., respectively (Table 1). This external electric field includes the impacts of the macroscopic reaction field and specific solute–solvent interactions [42]. These impacts cannot be separated in this approximation.

The impacts of the macroscopic reaction field can be accounted for using the PCM approximation. In this case we applied the external electric field to the OH-form only. This field simulates the difference of the specific solute–solvent interactions in the case of the NH- and OH-forms. The experimental values of the tautomeric equilibrium constant in cyclohexane and chloroform can be reproduced at a field strength of 0.0030 a.u. and 0.0023 a.u., respectively (Table 1). The search for the best values of the external electric field strength is simplified by the almost linear dependence of the energy on the field strength in all cases considered (Table S16 in the Supporting Information).

What does it mean that the field strengths required to simulate the effect of the specific solute–solvent interactions in cyclohexane and chloroform are large, and the value in cyclohexane is slightly larger than that in chloroform? This may mean that the PCM approximation underestimates the stabilization of the OH-form in nonpolar solvents in general and that in cyclohexane the error is larger than in chloroform. Alternatively, this may mean that the difference between the stabilization of the OH- and NH-forms due to specific interactions in cyclohexane is greater than in chloroform. Besides solute–solvent interactions, it could be a self-aggregation in the OH-form or the effect of impurities [71,72]. A more rigorous and complete answer to this question is of great interest, but cannot be given here, since it requires high-precision experimental measurements. We can only estimate the impacts of the macroscopic reaction field of cyclohexane and chloroform and the specific interactions in these solvents in terms of the external field strength. The impacts of the macroscopic reaction field of cyclohexane and chloroform will be called S^cy^ and S^ch^, respectively. The impacts of the specific solute–solvent interactions in cyclohexane and chloroform will be called I^cy^ and I^ch^. It follows from the PCM approximation that I^cy^ = 0.0030 a.u. and I^ch^ = 0.0023 a.u. When the gas-phase approximation is used, the field strengths are S^cy^ − I^cy^ = 0.0007 a.u. and S^ch^ − I^ch^ = 0.0023 a.u. The negative sign is used to give I and S as positive. Therefore, the impacts of the macroscopic reaction field of cyclohexane and chloroform are S^cy^ = 0.0037 a.u. and S^ch^ = 0.0046 a.u. The fact that S^cy^ < S^ch^ is consistent with the higher polarity of chloroform. The nature of the relatively large impacts of I^cy^ and I^ch^ are yet to be understood.

## 3. Materials and Methods

The Gaussian 09.D.01 program package was used for the density functional theory (DFT) quantum chemical calculations [73]. Geometry optimizations were done using the *ω*B97XD density functional theory (DFT) hybrid functional [74,75]. These calculations were done using the Def2QZVP basis set for the alkali metal halides and H_3_N···HF and the Def2TZVP basis set for pyridone and hydroxypyridine [76,77]. For LiAt, some calculations were also performed using the Def2QZV basis set. The parameters for SMD calculations were adapted from the Minnesota Solvent Descriptor Database [78]. Cyclohexane: Eps = 2.0165, EpsInf = 2.0352, HbondAcidity = 0.00, HbondBasicity = 0.00, SurfaceTensionAtInterface = 35.48, CarbonAromaticity = 0.0, ElectronegativeHalogenicity = 0.00. Chloroform: Eps = 4.7113, EpsInf = 2.0963, HbondAcidity = 0.15, HbondBasicity = 0.02, SurfaceTensionAtInterface = 38.39, CarbonAromaticity = 0.0, ElectronegativeHalogenicity = 0.75.

The combined use of an external electric field and the PСM or SMD approximations has a specific feature for alkali metal halides. The potential energy of a dipole in an external electric field is U = −q·R·E, where q stands for the charges, R stands for the distance between them, and E stands for the strength of the electric field. When R →∞, U →−∞ for any E > 0. Consequently, an M^+^···X^−^ molecule is a local minimum in this approximation, while the global minimum corresponds to a dissociated state. This local minimum does not show imaginary vibrational frequencies.

It was determined empirically that the dielectric dependence of molecular properties could be closely fit using the Kirkwood–Onsager parameter (ε − 1)/(ε + 0.5), where ε stands for the dielectric constant [79]. This parameter is used in this work to demonstrate the dielectric dependence of M···X distances.

Alkali metal halide dissociation energies at different conditions were calculated as a difference between the electronic energy of a given alkali metal halide complex and the sums of the electronic energies of the alkali metal and halogen atoms or the alkali metal cations and halogen anions at the same conditions.

## 4. Conclusions

The AuF approach models solvent [42,43] and matrix [56] effects using an external electric field applied to the molecular system under study. In this research, we report how the modeling results change in connection with the PCM approximation and with the use of the AuF approach to nonpolar solvents.

Note that this external electric field is fictitious in nature. It is just a tool to put pressure on the electron density of the molecule under investigation in order to model the changes caused by multiple interactions with solvent molecules. Therefore, this fictitious external electric field should not be confused with real local electric fields present in electrolytes [80] or real external electric fields applied to change physical [81,82] and chemical [83,84] properties of solutions. At the same time, the strength of this fictitious field is only about one order of magnitude smaller than that of these real electric fields.

It is shown that the AuF approach can simulate the dissociation of alkali metal halides. For small perturbation of these molecules, the use of the PCM approximation is optional. In contrast, for large disturbances and especially close to the dissociation it is strongly recommended to use the PCM approximation. Similar conclusions can be drawn for proton transfer in H_3_N···HF. For the hydrogen bond of the molecular type, H_3_N···HF, the difference between the results obtained in the gas-phase and PCM approximations is small. For the hydrogen bond of the ionic type, [H_3_NH]^+^···F^−^, the difference becomes much larger.

The AuF approach may be used to model solvent effects in nonpolar solvents. The integral effect can be estimated using the gas-phase approximation. By comparing these results with those obtained in the PCM approximation, we can distinguish between the effects of the macroscopic reaction field of the solvent and the specific solute–solvent interactions. In this work, such an analysis was carried out using experimental data on tautomeric equilibria between pyridone and hydroxypyridine in cyclohexane and chloroform. Although this analysis cannot determine the nature of the interactions taking place in the solution, it still allows us to evaluate the influence of these interactions on the parameters of the system under study and the need to take them into account when interpreting experimental data.

## Figures and Tables

**Table 1:** Electronic energy differences between the NH- and OH-forms of 2,6-di-tert-butyl-4-hydroxy-pyridine—experimental values and those calculated under different approximations.

Model	E(NH)–E(OH), a.u.
Exp. in cyclohexane [57]	0.0020
Exp. in chloroform [57]	−0.0021
Gas-phase DFT	0.0036
PCM cyclohexane	−0.0007
PCM chloroform	−0.0043
SMD cyclohexane	−0.0017
SMD chloroform	−0.0053
Field 0.0007 a.u.	0.0019
Field 0.0023 a.u.	−0.0019
PCM cyclohexane and Field 0.0030 a.u. on the OH-form	0.0021
PCM chloroform and Field 0.0023 a.u. on the OH-form	−0.0021

## Data Availability

No new data were created or analyzed in this study. Data sharing is not applicable to this article.

## Supplementary Materials

The following are available online. Alkali metal···halogen distance in KBr, CsF, and CsAt, as a function of (ε − 1)/(ε + 0.5), basis set dependence of this function for LiAt, correlations between the external electric fields required to obtain the same alkali metal···halogen distance in LiF, KBr, and LiAt under different approximations, numerical values of the calculated parameters presented in the figures.

## Sample Availability

Samples of the compounds are not available from the authors.

## References

[1] Řezáč J. (2020). Non-covalent interactions atlas benchmark data sets: Hydrogen bonding. J. Chem. Theory Comput..

[2] Bartashevich E., Matveychuk Y., Tsirelson V. (2019). Identification of the tetrel bonds between halide anions and carbon atom of methyl groups using electronic criterion. Molecules.

[3] Del Bene J.E., Alkorta I., Elguero J. (2019). N…C and S…S interactions in complexes, molecules, and transition structures HN(CH)SX:SCO, for X = F, Cl, NC, CCH, H, and CN. Molecules.

[4] Sagan F., Mitoraj M.P. (2019). Kinetic and Potential energy contributions to a chemical bond from the charge and energy decomposition scheme of extended transition state natural orbitals for chemical valence. J. Phys. Chem. A.

[5] Ostras A.S., Ivanov D.M., Novikov A.S., Tolstoy P.M. (2020). Phosphine oxides as spectroscopic halogen bond descriptors: IR and NMR correlations with interatomic distances and complexation energy. Molecules.

[6] Michalczyk M., Zierkiewicz W., Wysokiński R., Scheiner S. (2019). Theoretical studies of IR and NMR spectral changes induced by sigma-hole hydrogen, halogen, chalcogen, pnicogen, and tetrel bonds in a model protein environment. Molecules.

[7] Panek J.J., Jezierska A. (2018). N-oxide Derivatives: Car-Parrinello molecular dynamics and electron localization function study on intrarnolecular hydrogen bonds. J. Phys. Chem. A.

[8] Grabowski S. (2020). Hydrogen bond and other Lewis acid—Lewis base interactions as preliminary stages of chemical reactions. Molecules.

[9] Borštnar R., Repič M., Kamerlin S.C.L., Vianello R., Mavri J. (2012). Computational study of the pKa values of potential catalytic residues in the active site of monoamine oxidase, B. J. Chem. Theory Comput..

[10] Lakshmipriya A., Chaudhary M., Mogurampelly S., Klein M.L., Suryaprakash N. (2018). Intramolecular hydrogen bonding appetency for conformational penchants in oxalohydrazide fluoro derivatives: NMR, MD, QTAIM, and NCI studies. J. Phys. Chem. A.

[11] Bauzá A., Alkorta I., Frontera A., Elguero J. (2013). On the reliability of pure and hybrid DFT methods for the evaluation of halogen, chalcogen, and pnicogen bonds involving anionic and neutral electron donors. J. Chem. Theory Comput..

[12] Alkorta I., Sánchez-Sanz G., Elguero J., Del Bene J.E. (2012). Influence of hydrogen bonds on the P···P pnicogen bond. J. Chem. Theory Comput..

[13] Nenajdenko V.G., Shikhaliyev N.G., Maharramov A.M., Bagirova K.N., Suleymanova G.T., Novikov A.S., Khrustalev V.N., Tskhovrebov A.G. (2020). Halogenated diazabutadiene dyes: Synthesis, structures, supramolecular features, and theoretical studies. Molecules.

[14] Jóźwiak K., Jezierska A., Panek J.J., Goremychkin E.A., Tolstoy P.M., Shenderovich I.G., Filarowski A. (2020). Inter- vs. intramolecular hydrogen bond patterns and proton dynamics in nitrophthalic acid associates. Molecules.

[15] Panek J.J., Jezierska A. (2007). Symmetry-adapted perturbation theory analysis of the N···HX hydrogen bonds. J. Phys. Chem. A.

[16] Golubev N.S., Melikova S.M., Shchepkin D.N., Shenderovich I.G., Tolstoy P.M., Denisov G.S. (2003). Interpretation of H/D isotope effects on NMR chemical shifts of [FHF]^−^ ion based on calculations of nuclear magnetic shielding tensor surface. Z. Phys. Chem..

[17] Mahmoudi G., Afkhami F.A., Kennedy A.R., Zubkov F.I., Zangrando E., Kirillov A.M., Molins E., Mitoraj M.P., Safin D.A. (2020). Lead(II) coordination polymers driven by pyridine-hydrazine donors: From anion-guided self-assembly to structural features. Dalton Trans..

[18] Arp F.F., Bhuvanesh N., Blümel J. (2019). Hydrogen peroxide adducts of triarylphosphine oxides. Dalton Trans..

[19] Tupikina E.Y., Bodensteiner M., Tolstoy P.M., Denisov G.S., Shenderovich I.G. (2018). P = O moiety as an ambidextrous hydrogen bond acceptor. J. Phys. Chem. C.

[20] Vener M.V., Shishkina A.V., Rykounov A.A., Tsirelson V.G. (2013). Cl··· Cl interactions in molecular crystals: Insights from the theoretical charge density analysis. J. Phys. Chem. A.

[21] Ahn S.H., Cluff K.J., Bhuvanesh N., Blümel J. (2015). Hydrogen peroxide and di(hydroperoxy)propane adducts of phosphine oxides as stoichiometric and soluble oxidizing agents. Angew. Chem. Int. Ed..

[22] Shenderovich I.G. (2013). Effect of non-covalent interactions on the ^31^P chemical shift tensor of phosphine oxides, phosphinic, phosphonic, and phosphoric acids and their complexes with Lead(II). J. Phys. Chem. C.

[23] Palusiak M., Grabowski S.J. (2008). Do intramolecular halogen bonds exist? Ab initio calculations and crystal structures’ evidences. Struct. Chem..

[24] Sobczyk L., Grabowski S.J., Krygowski T.M. (2005). Interrelation between H-bond and Pi-electron delocalization. Chem. Rev..

[25] Limbach H.-H., Chan-Huot M., Sharif S., Tolstoy P.M., Shenderovich I.G., Denisov G.S. (2011). Critical hydrogen bonds and protonation states of pyridoxal 5’-phosphate revealed by NMR. Biochim. Biophys. Acta.

[26] Lesnichin S.B., Tolstoy P.M., Limbach H.-H., Shenderovich I.G. (2010). Counteranion-dependent mechanisms of intramolecular proton transfer in aprotic solution. Phys. Chem. Chem. Phys..

[27] Gurinov A.A., Rozhkova Y.A., Zukal A., Čejka J., Shenderovich I.G. (2011). Mutable Lewis and Brønsted acidity of aluminated SBA-15 as revealed by NMR of adsorbed pyridine−^15^N. Langmuir.

[28] Gedat E., Schreiber A., Findenegg G.H., Shenderovich I., Limbach H.-H., Buntkowsky G. (2001). Stray field gradient NMR reveals effects of hydrogen bonding on diffusion coefficients of pyridine in mesoporous silica. Magn. Reson. Chem..

[29] Orozco M., Luque F.J. (2000). Theoretical methods for the description of the solvent effect in biomolecular systems. Chem. Rev..

[30] Benighaus T., Thiel W. (2009). A general boundary potential for hybrid QM/MM simulations of solvated biomolecular systems. J. Chem. Theory Comput..

[31] Pylaeva S., Allolio C., Koeppe B., Denisov G.S., Limbach H.-H., Sebastiani D., Tolstoy P.M. (2015). Proton transfer in a short hydrogen bond caused by solvation shell fluctuations: An ab initio MD and NMR/UV study of an (OHO)−bonded system. Phys. Chem. Chem. Phys..

[32] Onufriev O. (2008). Implicit solvent models in molecular dynamics simulations: A brief overview. Annu. Rep. Comput. Chem..

[33] Clabaut P., Schweitzer B., Götz A.W., Michel C., Steinmann S.N. (2020). Solvation free energies and adsorption energies at the metal/water interface from hybrid quantum-mechanical/molecular mechanics simulations. J. Chem. Theory Comput..

[34] Vianello R., Domene C., Mavri J. (2016). The use of multiscale molecular simulations in understanding a relationship between the structure and function of biological systems of the brain: The application to monoamine oxidase enzymes. Front. Neurosci..

[35] Cossi M., Barone V., Cammi R., Tomasi J. (1996). Ab initio study of solvated molecules: A new implementation of the polarizable continuum model. Chem. Phys. Lett..

[36] Tomasi J., Mennucci B., Cammi R. (2005). Quantum mechanical continuum solvation models. Chem. Rev..

[37] Scalmani G., Frisch M.J. (2010). Continuous surface charge polarizable continuum models of solvation. I. General formalism. J. Chem. Phys..

[38] Marenich A.V., Cramer C.J., Truhlar D.G. (2009). Universal solvation model based on solute electron density and on a continuum model of the solvent defined by the bulk dielectric constant and atomic surface tensions. J. Phys. Chem. B.

[39] Gurinov A.A., Denisov G.S., Borissova A.O., Goloveshkin A.S., Greindl J., Limbach H.-H., Shenderovich I.G. (2017). NMR study of solvation effect on the geometry of proton-bound homodimers of increasing size. J. Phys. Chem. A.

[40] Del Bene J.E., Bartlett R.J., Elguero J. (2002). Interpreting ^2h^J(F,N), ^1h^J(H,N) and ^1J^(F,H) in the hydrogen-bonded FH–collidine complex. Magn. Reson. Chem..

[41] Shenderovich I.G. (2018). Simplified calculation approaches designed to reproduce the geometry of hydrogen bonds in molecular complexes in aprotic solvents. J. Chem. Phys..

[42] Shenderovich I.G., Denisov G.S. (2019). Solvent effects on acid-base complexes. What is more important: A macroscopic reaction field or solute-solvent interactions?. J. Chem. Phys..

[43] Shenderovich I.G., Denisov G.S. (2020). Adduct under field—a qualitative approach to account for solvent effect on hydrogen bonding. Molecules.

[44] Dominikowska J., Palusiak M. (2018). Tuning aromaticity of para-substituted benzene derivatives with an external electric field. ChemPhysChem.

[45] Liang H., Chai B., Chen G., Chen W., Chen S., Xiao H., Lin S. (2015). Electric field-driven acid-base transformation: Proton transfer from acid (HBr/HF) to base (NH_3_/H_2_O). Chem. Res. Chin. Univ..

[46] Astrakas L., Gousias C., Tzaphlidou M. (2011). Electric field effects on chignolin conformation. J. Appl. Phys..

[47] Alkorta I., Elguero J., Provasi P.F., Pagola G.I., Ferraro M.B. (2011). Electric field effects on nuclear magnetic shielding of the 1:1 and 2:1 (homo and heterochiral) complexes of XOOX′ (X, X′ = H, CH_3_) with lithium cation and their chiral discrimination. J. Chem. Phys..

[48] Mata I., Alkorta I., Espinosa E., Molins E. (2011). Relationships between interaction energy, intermolecular distance and electron density properties in hydrogen bonded complexes under external electric fields. Chem. Phys. Lett..

[49] Mata I., Molins E., Alkorta I., Espinosa E. (2009). Effect of an external electric field on the dissociation energy and the electron density properties: The case of the hydrogen bonded dimer HF⋯HF. J. Chem. Phys..

[50] Del Bene J.E., Jordan M.J.T. (2002). To what extent do external fields and vibrational and isotopic effects influence NMR coupling constants across hydrogen bonds? Two-bond Cl-N spin-spin coupling constants (^2h^JCl-N) in model ClH:NH_3_ complexes. J. Phys. Chem. A.

[51] Bevitt J., Chapman K., Crittenden D., Jordan M.J.T., Del Bene J.E. (2001). An ab initio study of anharmonicity and field effects in hydrogen-bonded complexes of the deuterated analogues of HCl and HBr with NH_3_ and N(CH_3_). J. Phys. Chem. A.

[52] Jordan M.J.T., Del Bene J.E. (2000). Unraveling environmental effects on hydrogen-bonded complexes: Matrix effects on the structures and proton-stretching frequencies of hydrogen-halide complexes with ammonia and trimethylamine. J. Am. Chem. Soc..

[53] Zhan C.-G., Chipman D.M. (1999). Reaction field effects on nitrogen shielding. J. Chem. Phys..

[54] Ramos M., Alkorta I., Elguero J., Golubev N.S., Denisov G.S., Benedict H., Limbach H.-H. (1997). Theoretical study of the influence of electric fields on hydrogen-bonded acid−base complexes. J. Phys. Chem. A.

[55] Suydam I.T., Snow C.D., Pande V.S., Boxer S.G. (2006). Electric fields at the active site of an enzyme: Direct comparison of experiment with theory. Science.

[56] Shenderovich I.G. (2020). Electric field effect on ^31^P NMR magnetic shielding. J. Chem. Phys..

[57] Frank J., Katritzky A.R. (1976). Tautomeric pyridines. Part XV. Pyridone–hydroxypyridine equilibria in solvents of differing polarity. J. Chem. Soc. Perkin Trans..

[58] Wang Z., Danovich D., Ramanan R., Shaik S. (2018). Oriented-external electric fields create absolute enantioselectivity in Diels–Alder reactions: Importance of the molecular dipole moment. J. Am. Chem. Soc..

[59] Chranina O.V., Czerniakowski F.P., Denisov G.S. (1988). UV-Vis electrochromism due to proton-transfer. J. Mol. Struct..

[60] Bergmann K., Eigen M., de Maeyer L. (1963). Dielektrische absorption als folge chemischer relaxation. Ber. Bunsenges. Phys. Chem..

[61] Steiner T. (2002). The hydrogen bond in the solid state. Angew. Chem. Int. Ed..

[62] Steiner T., Saenger W. (1992). Covalent bond lengthening in hydroxyl groups involved in three-center and in cooperative hydrogen bonds. Analysis of low-temperature neutron diffraction data. J. Am. Chem. Soc..

[63] Kong S., Borissova A.O., Lesnichin S.B., Hartl M., Daemen L.L., Eckert J., Antipin M.Y., Shenderovich I.G. (2011). Geometry and spectral properties of the protonated homodimer of pyridine in the liquid and solid states. A combined NMR, X-ray diffraction and inelastic neutron scattering study. J. Phys. Chem. A.

[64] Grabowski S.J. (2000). The bond valence model in analysing H-bonds of crystal structures. J. Mol. Struct..

[65] Del Bene J.E., Alkorta I., Elguero J. (2008). A Systematic comparison of second-order polarization propagator approximation (SOPPA) and equation-of-motion coupled cluster singles and doubles (EOM-CCSD) spin-spin coupling constants for selected singly bonded molecules, and the hydrides NH_3_, H_2_O, and HF and their protonated and deprotonated ions and hydrogen-bonded complexes. J. Chem. Theory Comput..

[66] Biczysko M., Latajka Z. (1999). The influence of water molecules on the proton position in H_3_N ‒ HX (X = F, Cl, Br) complexes. Chem. Phys. Lett..

[67] Tsuchida N., Yamabe S. (2005). Reaction paths of tautomerization between hydroxypyridines and pyridones. J. Phys. Chem. A.

[68] Elguero J., Katritzky A.R., Denisko O.V. (2000). Prototropic tautomerism of heterocycles: Heteroaromatic tautomerism-General overview and methodology. Adv. Heterocycl. Chem..

[69] Luque F.J., Zhang Y., Alemán C., Bachs M., Gao J., Orozco M. (1996). Solvent effects in chloroform solution: Parametrization of the MST/SCRF continuum model. J. Phys. Chem..

[70] Gao J., Shao L. (1994). Polarization effects on the tautomeric equilibria of 2- and 4-hydroxypyridine in aqueous and organic solution. J. Phys. Chem..

[71] Andreeva D.V., Ip B., Gurinov A.A., Tolstoy P.M., Denisov G.S., Shenderovich I.G., Limbach H.-H. (2006). Geometrical features of hydrogen bonded complexes involving sterically hindered pyridines. J. Phys. Chem. A.

[72] Shenderovich I.G. (2019). The partner does matter: The structure of heteroaggregates of acridine orange in water. Molecules.

[73] Frisch M.J., Trucks G.W., Schlegel H.B., Scuseria G.E., Robb M.A., Cheeseman J.R., Scalmani G., Barone V., Mennucci B., Petersson G.A. (2013). Gaussian 09, Revision, D.01.

[74] Chai J.-D., Head-Gordon M. (2008). Long-range corrected hybrid density functionals with damped atom-atom dispersion corrections. Phys. Chem. Chem. Phys..

[75] Grimme S. (2006). Semiempirical GGA-type density functional constructed with a long-range dispersion correction. J. Comput. Chem..

[76] Weigend F., Ahlrichs R. (2005). Balanced basis sets of split valence, triple zeta valence and quadruple zeta valence quality for H to Rn: Design and assessment of accuracy. Phys. Chem. Chem. Phys..

[77] Pritchard B.P., Altarawy D., Didier B., Gibson T.D., Windus T.L. (2019). A new basis set exchange: An open, up-to-date resource for the molecular sciences community. J. Chem. Inf. Model..

[78] Winget P., Dolney D.M., Giesen D.J., Cramer C.J., Truhlar D.G. Minnesota Solvent Descriptor Database. http://comp.chem.umn.edu/solvation/mnsddb.pdf.

[79] Onsager L. (1936). Electric moments of molecules in liquids. J. Am. Chem. Soc..

[80] Sellner B., Valiev M., Kathmann S.M. (2013). Charge and electric field fluctuations in aqueous NaCl electrolytes. J. Phys. Chem. B.

[81] Nardo V.M., Cassone G., Ponterio R.C., Saija F., Sponer J., Tommasini M., Trusso S. (2020). Electric-field-induced effects on the dipole moment and vibrational modes of the centrosymmetric indigo molecule. J. Phys. Chem. A.

[82] Cassone G., Sponer J., Trusso S., Saija F. (2019). Ab initio spectroscopy of water under electric fields. Phys. Chem. Chem. Phys..

[83] Cassone G. (2020). Nuclear quantum effects largely influence molecular dissociation and proton transfer in liquid water under an electric field. J. Phys. Chem. Lett..

[84] Cassone G., Sofia A., Rinaldi G., Sponer J. (2019). Catalyst-free hydrogen synthesis from liquid ethanol: An ab initio molecular dynamics study. J. Phys. Chem. C.

