# Supplementary material for: Modeling of Solute-Solvent Interactions Using an External Electric Field—From Tautomeric Equilibrium in Nonpolar Solvents to the Dissociation of Alkali Metal Halides
Source: Molecules. 2021 Feb 26;26(5):1283. doi: 10.3390/molecules26051283 (PMC7956811; doi:10.3390/molecules26051283)
Supplement: Supplementary file 1 [file molecules-26-01283-s001.pdf]

molecules

Supporting Information for Publication

Modeling of solute-solvent interactions using an external electric field - from tautomeric equilibrium in nonpolar solvents to the dissociation of alkali metal halides.

Ilya G. Shenderovich[1,*] and Gleb S. Denisov[2]

[1]Institute of Organic Chemistry, University of Regensburg, Universitaetstrasse 31, 93053 Regensburg Germany; Ilya.Shenderovich@ur.de
[2]Department of Physics, St.Petersburg State University, 198504 St. Petersburg, Russian Federation; gldenisov@yandex.ru
* Correspondence: Ilya.Shenderovich@ur.de

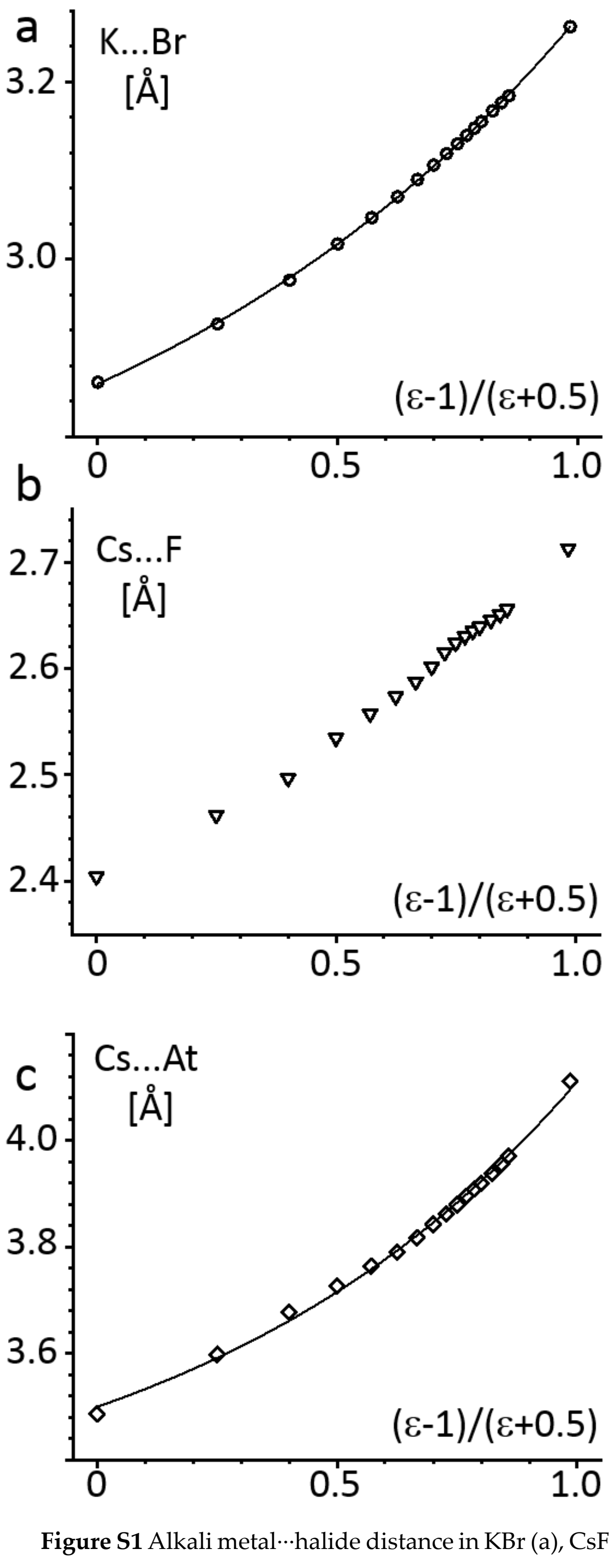

Figure S1 Alkali metal···halide distance in KBr (a), CsF (b), and CsAt (c) as a function of the Kirkwood-Onsager parameter (ε-1)/(ε+0.5) under the PCM approximation at 1 ≤ ε = ≤ 100 at the *ω*B97XD/ Def2QZVP level of theory.

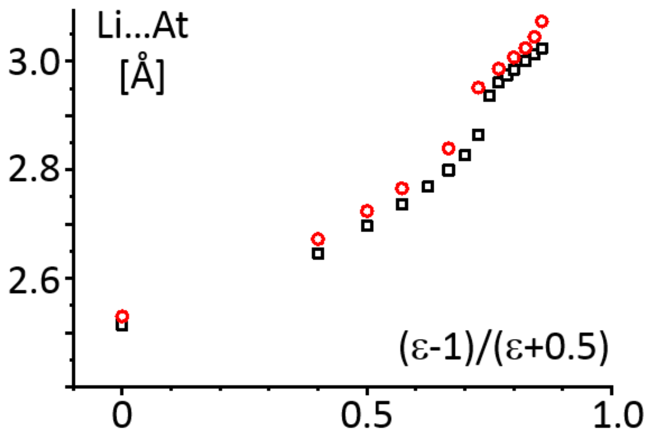

Figure S2 (a) Li···At distance as a function of the Kirkwood-Onsager parameter (ε-1)/(ε+0.5) under the PCM approximation at 1 ≤ ε = ≤ 10 at the ωB97XD/ Def2QZVP (empty black squares) and ωB97XD/ Def2QZV (empty red cycles) levels of theory.

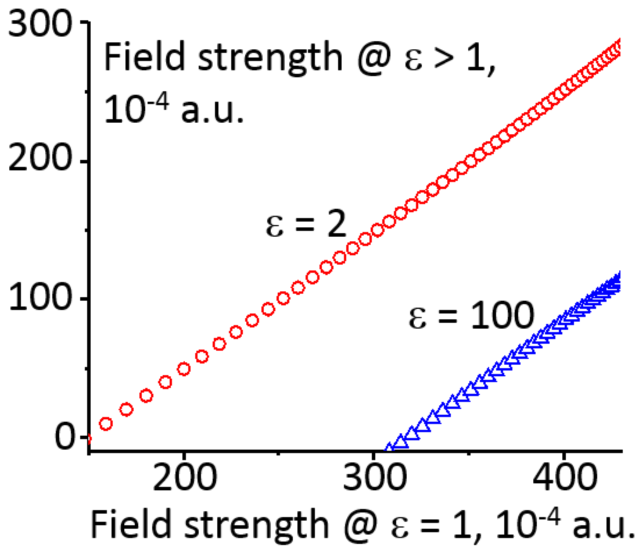

Figure S3 A correlation between the external electric fields required to obtain the same alkali metal···halide distance in LiF in the gas phase (ε = 1) and PCM ((ε = 2 and 100) approximations at the distance smaller than 1.90 Å.

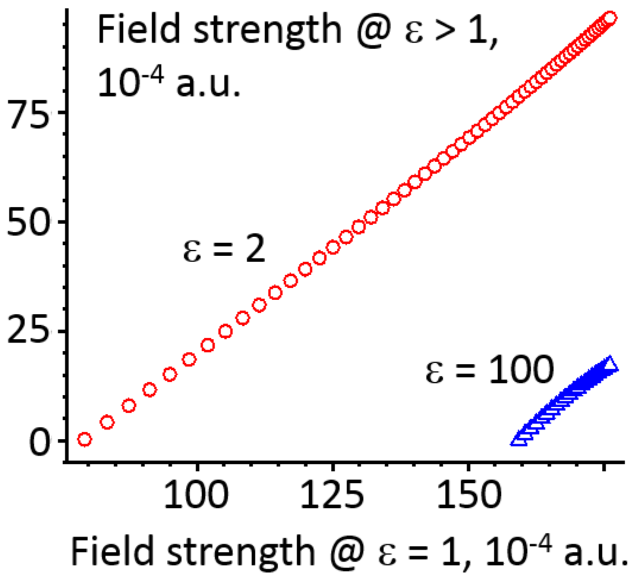

Figure S4 A correlation between the external electric fields required to obtain the same alkali metal···halide distance in KBr in the gas phase (ε = 1) and PCM (ε = 2 and 100) approximations at the distance smaller than 3.45 Å.

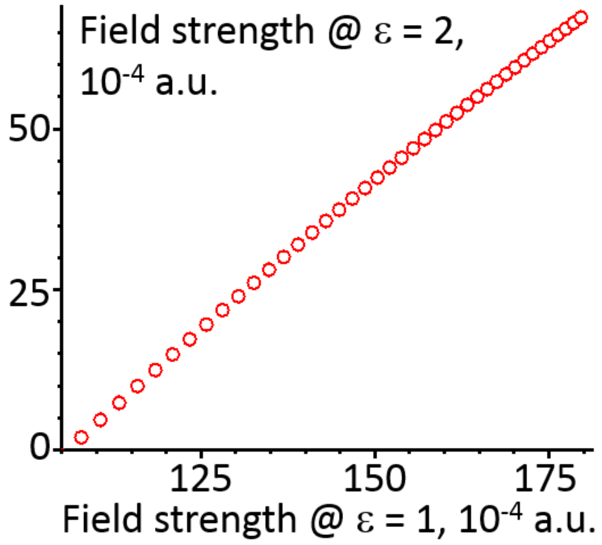

Figure S5 A correlation between the external electric fields required to obtain the same alkali metal···halide distance in LiAt in the gas phase (ε = 1) and PCM (ε = 2) approximations at the distance smaller than 2.85 Å.

Table S1. Alkali metal···halide distances [Å] and electronic energies [a.u.] (*ω*B97XD/Def2QZVP) under the gas-phase approximation (ε = 1).

	F	Cl	Br	I	At
Li	1.5795<br>-107.4509707	2.043<br>-467.8279064	2.2001<br>-2581.8733042	2.4226<br>-305.3879137	2.5146<br>-270.161927
Na	1.9453<br>-262.1981689	2.3893<br>-622.5889409	2.5371<br>-2736.6373698	2.7520<br>-460.1553744	2.8386<br>-424.9307579
K	2.1885<br>-699.8560827	2.6950<br>-1060.2465585	2.8611<br>-3174.2951829	3.0883<br>-897.8135439	3.1819<br>-862.5889203
Rb	2.3395<br>-124.0251425	2.8599<br>-484.4183932	3.0244<br>-2598.467571	3.2576<br>-321.9868547	3.3492<br>-286.7625102
Cs	2.4045<br>-120.0958682	2.9704<br>-480.4859365	3.1460<br>-2594.5349468	3.3957<br>-318.0543119	3.4858<br>-282.830071

Table S2. Alkali metal···halide distances [Å] and electronic energies [a.u.] (*ω*B97XD/Def2QZVP) under the PCM approximation (ε = 2).

	F	Cl	Br	I	At
Li	1.6350<br>-107.4769802	2.1359<br>-467.8561941	2.3064<br>-2581.9016974	2.5447<br>-305.4165387	2.6462<br>-270.1899745
Na	2.0107<br>-262.2268962	2.4949<br>-622.6179402	2.6578<br>-2736.6658393	2.8989<br>-460.1832689	2.9974<br>-424.9576597
K	2.2716<br>-699.8785341	2.8071<br>-1060.2707732	2.9761<br>-3174.3192246	3.2254<br>-897.8373207	3.3168<br>-862.61198
Rb	2.4300<br>-124.0478772	2.9801<br>-484.4424922	3.1562<br>-2598.4914464	3.4069<br>-322.0102595	3.4989<br>-286.785148
Cs	2.4969<br>-120.1137279	3.1074<br>-480.5068103	3.3034<br>-2594.5560154	3.5713<br>-318.0752152	3.6766<br>-282.8504101

Table S3. Alkali metal···halide distances [Å] and electronic energies [a.u.] (ωB97XD/Def2QZVP) under the PCM approximation (ε = 100).

	F	Cl	Br	I	At
Li	1.7375<br>-107.5148924	2.4468<br>-467.9004281	2.6646<br>-2581.9481263	3.2980<br>-305.4658706	3.7768<br>-270.2396729
Na	2.1333<br>-262.2664955	2.7488<br>-622.6601798	2.9524<br>-2736.7084284	3.3876<br>-460.2269279	3.5844<br>-425.0009578
K	2.4191<br>-699.9112132	3.0506<br>-1060.3060771	3.2622<br>-3174.3546506	3.5837<br>-897.8728403	3.7134<br>-862.6468347
Rb	2.5970<br>-124.081146	3.2518<br>-484.4775591	3.4502<br>-2598.5264717	3.7751<br>-322.0449455	3.8902<br>-286.8190473
Cs	2.7127<br>-120.1425757	3.4316<br>-480.5392104	3.6582<br>-2594.5885918	3.9774<br>-318.107709	4.1113<br>-282.8821542

Table S4. Electronic energies [a.u.] (ωB97XD/Def2QZVP) of alkali metal and halogen atoms under the gas-phase (ε = 1) and PCM (ε = 2 and 100) approximations.

Atom	Dielectric constant		
	ε = 1	ε = 2	ε = 100
Li	-7.4907916	-7.5000192	-7.5124248
Na	-162.2704867	-162.2750283	-162.2807679
K	-599.9193014	-599.9221215	-599.9254953
Rb	-24.0953464	-24.0975498	-24.1001354
Cs	-20.1558066	-20.1577217	-20.1599369
F	-99.747555	-99.7477081	-99.7479578
Cl	-460.1606781	-460.1610328	-460.1616202
Br	-2574.2244953	-2574.2249059	-2574.2255897
I	-297.7608133	-297.7612895	-297.7620931
At	-262.546075	-262.5465294	-262.5472965

Table S5. Electronic energies [a.u.] (ωB97XD/Def2QZVP) of alkali metal cations and halogen anions under the gas-phase (ε = 1) and PCM (ε = 2 and 100) approximations.

Atom	Dielectric constant		
	$\varepsilon = 1$	$\varepsilon = 2$	$\varepsilon = 100$
Li^+	-7.2932287	-7.3913801	-7.4875687
Na^+	-162.0885102	-162.1692153	-162.2483082
K^+	-599.7680651	-599.8313017	-599.8932782
Rb^+	-23.9491455	-24.0077952	-24.0652783
Cs^+	-20.0198408	-20.0733449	-20.1257882
F^-	-99.862329	-99.932125	-100.0005525
Cl^-	-460.2921432	-460.3494947	-460.4058478
Br^-	-2574.3495923	-2574.4031334	-2574.4557595
I^-	-297.8790901	-297.9276439	-297.9755367
At^-	-262.6584922	-262.7043615	-262.7497369

Table S6. Alkali metal···halide distances [Å] (ωB97XD/Def2QZVP) under the PCM ($2.5 \leq \varepsilon \leq 10.0$, and $\varepsilon_{inf} = 2.0$) approximation.

ε	LiF	LiAt	KBr	CsF	CsAt
2.5	1.6501	2.6967	3.0171	2.5349	3.7261
3.0	1.6615	2.7368	3.0468	2.5575	3.7637
3.5	1.6703	2.7690	3.0704	2.5737	3.7906
4.0	1.6774	2.7994	3.0898	2.5875	3.817
4.5	1.6832	2.8268	3.1059	2.6016	3.8425
5.0	1.6879	2.8643	3.1190	2.6150	3.8623
5.5	1.6920	2.9368	3.1301	2.6240	3.8795
6.0	1.6954	2.9611	3.1396	2.6303	3.8953
6.5	1.6985	2.9749	3.1479	2.6351	3.9088
7.0	1.7011	2.9847	3.1552	2.6390	3.9197
8.0	1.7057	3.0006	3.1672	2.6455	3.938
9.0	1.7094	3.0131	3.1766	2.6509	3.9547
10.0	1.7124	3.0239	3.1842	2.6557	3.9707

Table S7. Li···At [Å] distances (ωB97XD/Def2QZV) under the PCM (1.0 ≤ ε ≤ 10.0) approximation.

ε	Li···At [Å]
1.0	2.5299
2.0	2.6724
2.5	2.7240
3.0	2.7659
4.0	2.8394
5.0	2.9516
6.0	2.9862
7.0	3.0073
8.0	3.0246
9.0	3.0452
10.0	3.0736

Table S8. The Li···F distance [Å] (ωB97XD/Def2QZVP) as a function of the external electric field under the gas-phase (ε = 1) and PCM (ε = 2 and 100) approximations.

Dielectric constant ε = 1		Dielectric constant ε = 2		Dielectric constant ε = 100	
Field [10^{-4} a.u.]	Distance [Å]	Field [10^{-4} a.u.]	Distance [Å]	Field [10^{-4} a.u.]	Distance [Å]
10	1.5827	25	1.6466	10	1.7461
50	1.5963	50	1.6593	20	1.7552
100	1.6149	75	1.6732	25	1.7600
150	1.6357	100	1.6885	30	1.7649
200	1.6595	200	1.7693	40	1.7753
225	1.6729	250	1.8321	45	1.7808
250	1.6873	270	1.8892	47	1.7830
275	1.7037	280	2.0550	49	1.7853
300	1.7232	290	2.0855	50	1.7865
325	1.7441	295	2.1013	60	1.7986
350	1.7680	297	2.1097	70	1.8118
400	1.9268	298	2.1149	74	1.8174
450	1.9783			75	1.8188
455	2.0181			80	1.8261
456	2.0330			90	1.8415
				100	1.8613
				130	2.1058
				131	2.1109
				132	2.1170

Table S9. The Li···At distance [Å] (ωB97XD/Def2QZVP) as a function of the external electric field under the gas-phase (ε = 1) and PCM (ε = 2 and 100) approximations.

Dielectric constant ε = 1		Dielectric constant ε = 2		Dielectric constant ε = 100	
Field [10^{-4} a.u.]	Distance [Å]	Field [10^{-4} a.u.]	Distance [Å]	Field [10^{-4} a.u.]	Distance [Å]
50	2.5662	25	2.7004	5	3.8820
70	2.5919	50	2.7691		
90	2.6215	75	2.9411		
95	2.6296	90	3.0229		
97	2.6330	95	3.1387		
98	2.6347	97	3.1473		
99	2.6364	98	3.1511		
100	2.6381				
150	2.7438				
200	2.9709				
210	3.0772				
215	3.1651				
217	3.2243				

Table S10. The K···Br distance [Å] (ωB97XD/Def2QZVP) as a function of the external electric field under the gas-phase (ε = 1) and PCM (ε = 2 and 100) approximations.

Dielectric constant ε = 1		Dielectric constant ε = 2		Dielectric constant ε = 100	
Field [10^{-4} a.u.]	Distance [Å]	Field [10^{-4} a.u.]	Distance [Å]	Field [10^{-4} a.u.]	Distance [Å]
10	2.8730	25	3.0406	5	3.3023
25	2.8914	50	3.1227	10	3.3394
50	2.9255	75	3.2317	13	3.3748
100	3.0268	80	3.2661	15	3.4066
125	3.0987	90	3.3554	17	3.4471
150	3.1965	95	3.4488	20	3.4998
175	3.4333	96	3.4711	21	3.5344
176	3.4485				
177	3.4743				
178	3.5494				

Table S11. The Cs···F distance [Å] (ωB97XD/Def2QZVP) as a function of the external electric field under the gas-phase (ε = 1) and PCM (ε = 2 and 100) approximations.

Dielectric constant ε = 1		Dielectric constant ε = 2		Dielectric constant ε = 100	
Field [10^{-4} a.u.]	Distance [Å]	Field [10^{-4} a.u.]	Distance [Å]	Field [10^{-4} a.u.]	Distance [Å]
10	2.4140	25	2.5479	50	3.0022
50	2.4606	50	2.5961	55	3.0439
100	2.5330	75	2.6530	56	3.1115
150	2.6301	100	2.7285		
175	2.6875	125	2.8216		
200	2.7584	130	2.8352		
225	2.9198	140	2.9665		
235	3.0246	143	3.0203		
		145	3.0355		
		146	3.0464		
		147	3.0666		

Table S12. The Cs···At distance [Å] (ωB97XD/Def2QZVP) as a function of the external electric field under the gas-phase (ε = 1) and PCM (ε = 2 and 100) approximations.

Dielectric constant ε = 1		Dielectric constant ε = 2		Dielectric constant ε = 100	
Field [10^{-4} a.u.]	Distance [Å]	Field [10^{-4} a.u.]	Distance [Å]	Field [10^{-4} a.u.]	Distance [Å]
10	3.5121	25	3.7845	10	4.2697
50	3.6389	50	4.0166	15	4.4131
100	3.9077	60	4.1826	17	4.5890
125	4.3145	63	4.2304		
127	4.4117	65	4.2737		
		68	4.3840		
		69	4.6153		

Table S13. The limiting values of the external electric field and the corresponding alkali metal···halide distances before the dissociation of the alkali metal halides under the gas-phase (ε = 1) and PCM (ε = 2 and 100) approximations at the *ω*B97XD/ Def2QZVP level of theory.

Complex	ε	Field [10^{-4} a.u.]	Distance [Å]
LiF	1	456	2.0330
LiF	2	298	2.1149
LiF	100	132	2.1170
LiAt	1	217	3.2243
LiAt	2	98	3.1511
LiAt	100	5	3.8820
KBr	1	178	3.5494
KBr	2	96	3.4711
KBr	100	21	3.5344
CsF	1	235	3.0246
CsF	2	147	3.0666
CsF	100	57	3.1115
CsAt	1	127	4.4117
CsAt	2	69	4.6153
CsAt	100	17	4.5890

Table S14. The $H_3N\cdots H$ and $N\cdots F$ distances [Å] of $H_3N\cdots HF$ as a function of the external electric field under the gas-phase (ε = 1) and PCM (ε = 2 and 100) approximations at the *ω*B97XD/ Def2QZVP level of theory.

Dielectric constant ε = 1			Dielectric constant ε = 2			Dielectric constant ε = 100		
Field [10^{-4} a.u.]	Distance [Å]		Field [10^{-4} a.u.]	Distance [Å]		Field [10^{-4} a.u.]	Distance [Å]	
	N···H	N···F		N···H	N···F		N···H	N···F
0	1.691455	2.643275	0	1.626176	2.591878	0	1.515280	2.513887
10	1.684053	2.637746	10	1.616979	2.585481	10	1.498524	2.504151
20	1.676486	2.632132	20	1.607515	2.578996	20	1.479760	2.493704
30	1.668771	2.626462	30	1.597761	2.572420	30	1.457992	2.482210
40	1.660954	2.620782	40	1.587677	2.565736	40	1.431423	2.469160
50	1.653063	2.615118	50	1.577243	2.558951	50	1.393095	2.452424
60	1.645114	2.609498	60	1.566412	2.552054	53	1.376025	2.445830
70	1.637098	2.603918	70	1.555106	2.545011	55	1.360802	2.440457
80	1.628920	2.598290	80	1.543184	2.537756	57	1.337685	2.433301
90	1.620565	2.592624	90	1.530501	2.530231	58	1.308655	2.426273
100	1.612003	2.586894	97	1.521083	2.524774	59	1.174877	2.440326
110	1.603129	2.581052	100	1.5169	2.5224	60	1.168097	2.444522
120	1.593986	2.575117	110	1.502173	2.514162	65	1.146102	2.462517
130	1.584580	2.569141	120	1.485838	2.505381	70	1.132006	2.478524
140	1.574785	2.563002	130	1.467460	2.495943	80	1.112544	2.509025
150	1.564624	2.556771	140	1.446026	2.485561	90	1.098415	2.540168
160	1.554004	2.550398	150	1.419353	2.473668	100	1.087051	2.573573
170	1.542805	2.543823	160	1.382073	2.459169	110	1.076688	2.613725
180	1.530909	2.537004	165	1.353878	2.450073	120	1.066838	2.665050
190	1.518206	2.529928	169	1.316054	2.441006	125	1.062538	2.693558
200	1.504457	2.522479	170	1.299472	2.438376	130	1.056512	2.741290
210	1.489425	2.514618	171	1.276582	2.436346	135	1.049115	2.820156
220	1.472719	2.506274	172	1.249375	2.436841	136	1.047039	2.848231
230	1.453624	2.497216	173	1.228109	2.439960			
240	1.430959	2.487197	175	1.203170	2.447515			
250	1.4024	2.4758	180	1.172130	2.465070			
260	1.361818	2.462349	190	1.141393	2.495904			
265	1.331101	2.454758	200	1.121996	2.526798			
269	1.294952	2.449472	210	1.106983	2.560446			
270	1.283955	2.448770	220	1.094237	2.599429			
271	1.272647	2.448592	230	1.081874	2.651445			
272	1.261444	2.449012	235	1.074742	2.690855			
275	1.232042	2.453386	240	1.067066	2.745126			
280	1.199619	2.465367	241	1.065121	2.761601			

290	1.163302	2.492365	242	1.063626	2.775048			
300	1.140431	2.521491	243	1.061978	2.791049			
320	1.109127	2.589412						
330	1.096162	2.634771						
335	1.090224	2.661318						
340	1.082799	2.701184						
345	1.073581	2.765841						
346	1.071238	2.786042						

Table S15. The H_3N···H and N···F distances [Å] of H_3N···HF under the gas-phase (ε = 1) and PCM (ε = 2 and 100) approximations at the *ω*B97XD/ Def2QZVP level of theory.

ε	N···H [Å]	N···F [Å]
1.0	1.691455	2.643275
2.0	1.626176	2.591878
2.5	1.609411	2.579269
3.0	1.597052	2.570134
4.0	1.580021	2.557800
5.0	1.568846	2.549869
6.0	1.560828	2.544271
7.0	1.554848	2.540141
8.0	1.550186	2.536949
9.0	1.546437	2.534402
10.0	1.543370	2.532328
100.0	1.515280	2.513887

Table S16. The electronic energies of the NH- and OH-forms of 2,6-Di-tert-butyl-4-hydroxy- calculated under different approximations at the *ω*B97XD/ Def2TZVP level of theory.

Model	E(NH) [a.u.]	E(OH) [a.u.]
Gas-phase DFT	-638.0649164	-638.0684887
PCM cyclohexane	-638.0715641	-638.0708409
PCM chloroform	-638.0772454	-638.0729736
SMD cyclohexane	-638.081364	-638.0796272
SMD chloroform	-638.0898505	-638.0845263
Field 0.0005 a.u.	-638.0663875	-638.0687791
Field 0.0007 a.u.	-638.0669894	-638.0689079
Field 0.0010 a.u.	-638.0679067	-638.0691151
Field 0.0023 a.u.	-638.0720824	-638.0702058
Field 0.0050 a.u.	-638.0817972	-638.0734741
PCM cyclohexane<br>Field 0.0005 a.u. for [OH]	-638.0715641	-638.0711734
PCM cyclohexane<br>Field 0.0010 a.u. for [OH]	-638.0715641	-638.0715583
PCM cyclohexane<br>Field 0.0030 a.u. for [OH]	-638.0715641	-638.0736249
PCM cyclohexane<br>Field 0.0038 a.u. for [OH]	-638.0715641	-638.0746883
PCM chloroform<br>Field 0.0005 a.u. for [OH]	-638.0772454	-638.0733486
PCM chloroform<br>Field 0.0010 a.u. for [OH]	-638.0772454	-638.0737821
PCM chloroform<br>Field 0.0023 a.u. for [OH]	-638.0772454	-638.0751839
PCM chloroform<br>Field 0.0025 a.u. for [OH]	-638.0772454	-638.0754348
